# Automatic vs. Manual Detection of High Frequency Oscillations in Intracranial Recordings From the Human Temporal Lobe

**DOI:** 10.3389/fneur.2020.563577

**Published:** 2020-10-19

**Authors:** Aljoscha Thomschewski, Nathalie Gerner, Patrick B. Langthaler, Eugen Trinka, Arne C. Bathke, Jürgen Fell, Yvonne Höller

**Affiliations:** ^1^Department of Neurology, Christian-Doppler Medical Center, Paracelsus Medical University, Salzburg, Austria; ^2^Department of Mathematics, Paris-Lodron University of Salzburg, Salzburg, Austria; ^3^Department of Psychology, Paris-Lodron University of Salzburg, Salzburg, Austria; ^4^Intelligent Data Analytics Lab Salzburg, Paris-Lodron University of Salzburg, Salzburg, Austria; ^5^Department of Epileptology, University Hospital Bonn, Bonn, Germany; ^6^Faculty of Psychology, University of Akureyri, Akureyri, Iceland

**Keywords:** high-frequency oscillations, visual memory, invasive EEG, electroencephalography, epilepsy

## Abstract

**Background:** High frequency oscillations (HFOs) have attracted great interest among neuroscientists and epileptologists in recent years. Not only has their occurrence been linked to epileptogenesis, but also to physiologic processes, such as memory consolidation. There are at least two big challenges for HFO research. First, detection, when performed manually, is time consuming and prone to rater biases, but when performed automatically, it is biased by artifacts mimicking HFOs. Second, distinguishing physiologic from pathologic HFOs in patients with epilepsy is problematic. Here we automatically and manually detected HFOs in intracranial EEGs (iEEG) of patients with epilepsy, recorded during a visual memory task in order to assess the feasibility of the different detection approaches to identify task-related ripples, supporting the physiologic nature of HFOs in the temporal lobe.

**Methods:** Ten patients with unclear seizure origin and bilaterally implanted macroelectrodes took part in a visual memory consolidation task. In addition to iEEG, scalp EEG, electrooculography (EOG), and facial electromyography (EMG) were recorded. iEEG channels contralateral to the suspected epileptogenic zone were inspected visually for HFOs. Furthermore, HFOs were marked automatically using an RMS detector and a Stockwell classifier. We compared the two detection approaches and assessed a possible link between task performance and HFO occurrence during encoding and retrieval trials.

**Results:** HFO occurrence rates were significantly lower when events were marked manually. The automatic detection algorithm was greatly biased by filter-artifacts. Surprisingly, EOG artifacts as seen on scalp electrodes appeared to be linked to many HFOs in the iEEG. Occurrence rates could not be associated to memory performance, and we were not able to detect strictly defined “clear” ripples.

**Conclusion:** Filtered graphoelements in the EEG are known to mimic HFOs and thus constitute a problem. So far, in invasive EEG recordings mostly technical artifacts and filtered epileptiform discharges have been considered as sources for these “false” HFOs. The data at hand suggests that even ocular artifacts might bias automatic detection in invasive recordings. Strict guidelines and standards for HFO detection are necessary in order to identify artifact-derived HFOs, especially in conditions when cognitive tasks might produce a high amount of artifacts.

## 1. Introduction

High frequency oscillations (HFOs) have gained considerable interest amongst neurologists and neuroscientists in the last decade. These relatively new electroencephalographic (EEG) markers are defined as single events of at least four oscillations with a frequency above 80 Hz that clearly stand out from the background EEG (1). Classically, HFOs have further been divided into two subgroups: ripples (80–250 Hz) and fast ripples (250–500 Hz; 2). Given these criteria, a high signal-to-noise ratio is key when attempting to detect HFOs. Hence, the first findings of HFOs stem from invasive EEG (iEEG) recordings with micro- or macroelectrodes (2–7).

As these recordings are only performed during presurgical evaluation in patients with drug resistant epilepsies, their occurrence has naturally been studied and linked to epilepsy and many findings indicate a link between HFOs and epileptogenity, both during ictal (8, 9) and interictal states (10–12). Besides there association with epilepsy, several studies also suggested an existence of a second HFO population, reflecting physiologic processes (3, 13–17). Especially entorhinal and hippocampal ripples have been associated with memory consolidation in animals (18, 19) and humans (20–23).

Albeit these numerous investigations, the detection of HFOs remains a highly debatable subject, and many aspects need to be considered. Besides technical considerations regarding the signal-to-noise ratio and data sampling (24–26), choosing the actual method of detection can be difficult. Considering the mentioned criteria (1), visual inspection requires enlarging the signal both in time scale and amplitude in order to discern these discrete events from the background EEG (27). Screening the data in such a way is highly time-consuming and visual detection can further be biased by the raters' subjective assessment of what “clearly stands out from the background EEG” (28, 29).

In contrast, automatic HFO detection is fast and objective. In facts, there exist a plethora of automatic detection algorithms for HFOs (30–34). Though considerably minimizing the time necessary to perform HFO detection, automatic detectors are prone to biases from signal artifacts (35–39), and they are seldom accurate on datasets they have not been trained on (24, 40). Furthermore, automatic detection algorithms are unable to differentiate between HFOs occurring as single elements and HFOs that are coupled with epileptiform discharges.

Given its more adaptive and strict results, manual detection may thus be necessary when dealing with data containing different (physiologic and pathologic) HFO populations and artifacts. For instance, when wanting to detect physiologic HFOs that are evoked by cognitive paradigms in patients with epilepsy. In the study at hand, we analyzed such a dataset. Using a dataset described by Axmacher et al. (20), we investigated stimulus-induced HFOs during encoding and retrieval to demonstrate possible differences between the two approaches of HFO detection, as well as to take advantage of the high sensitivity of automatic detectors and the specificity of a manual review when trying to link ripple occurrence to memory performance.

For this purpose, we assessed for both detection approaches: (1) whether ripple occurrence rates during encoding or retrieval phases differed between correct and incorrect responses in the memory task; (2) whether the event rates detected during encoding were predictive for the performance in the subsequent retrieval trials on a trial level; and (3) whether the amount of detected events was related to the response times in the memory task. We hypothesized the results to differ between automatically detected and manually detected events. Assuming that automatic detection results in less valid detections, we hypothesized that event rates revealed no or less of an association with memory performance as compared to events detected visually. Confirming our hypothesis would emphasize the importance for an accurate detection in order to differentiate physiologic, e.g., memory-related, from pathologic HFOs.

## 2. Methods

### 2.1. Subjects and Experimental Procedure

Ten patients with pharmacoresistant temporal lobe epilepsies (five women, mean age = 39.4 years, SD = 10.83), enrolled in a study that took place at the University Hospital Bonn between 2004 and 2006, were retrospectively analyzed. All patients received bilateral intracranial EEG (iEEG) recordings for presurgical evaluation. Patients enrolled in the study were asked to perform a visual memory task on a recording day previous to which no seizures had been experienced for 24 h. Detailed information on the patient sample may be found in Table 1. The study was approved by the local ethics committee, and all patients gave written informed consent before participating.

**Table 1:** Patient information.

**Subject**	**Age (years)**	**Gender**	**Structural lesion**	**Seizure type(s)**	**Onset age**	**SOZ (iEEG)**	**Further remarks**
P1	45	f	Hippocampal sclerosis right	Focal non-motor impaired awareness seizures	13	Right temporo-mesial	
P2	34	m	Hippocampal sclerosis right	Focal non-motor impaired awareness seizures + ftbTCS	n\a	Right temporo-mesial	
P3	54	m	Hippocampal sclerosis left	Focal aware non-motor seizures + focal motor impaired awareness seizures + ftbTCS	9	Left temporo-mesial	
P4	33	f	Hippocampal sclerosis + hypometabolism (FDG) temporal left	Focal non-motor impaired awareness seizures + ftbTCS	n\a	Left temporo-mesial	Left sided speech dominance (WADA)
P5	44	f	MRI negative; hypometabolism (FDG) temporo-polar left	Focal aware non-motor seizures	18	Left temporo-polar	Right handed
P6	46	m	Hippocampal sclerosis + hypometabolism (FDG) temporal mesial and polar left	Focal motor and non-motor impaired awareness seizures	11	Left temporo-mesial	Right handed, bilateral speech (WADA)
P7	47	m	Hippocampal sclerosis right; discrete hypometabolism (FDG) temporo-polar left	Focal aware motor seizures + ftbTCS	n\a	Right temporo-mesial	
P8	45	m	Hippocampal sclerosis and hypometabolism (FDG) temporal right	Focal non-motor impaired awareness seizures	6	Bitemporal	
P9	18	f	Hippocampal sclerosis left	Focal motor and non-motor impaired awareness seizures	n\a	Most prominently left temporo-mesial	Hint of right-sided hippocampal sclerosis
P10	28	f	Hippocampal sclerosis and temporo-polar dysplasia right	Focal motor impaired awareness seizures	n\a	Right temporo-mesial	Ictal aphasia

*SOZ, seizure onset zone; iEEG, intracranial electroencephalography; f, female; m, male; ftbTCS, focal to bilateral tonic clonic seizures; FDG, fluorodeoxyglucose; MRI, magnetic resonance imaging*.

The visual working memory task contained two encoding as well as one retrieval phase, intertwined by a nap time. During encoding, patients were presented with 80 pictures of either landscapes or houses. Each image was presented for 1,200 ms with a variable interstimulus interval of 1,800 ± 200 ms. In order to ensure that patients stayed focused they were asked to indicate via button press whether they saw an image depicting a house or landscape. After this initial encoding phase, patients were asked to rest in a darkened room for 60 min and try to nap. Following a pause of 15 min after this period of resting there was another encoding phase with 80 novel images. After another break of 15 min, patients were presented with all 160 images they had learned previously plus an addition of 80 unlearned images. During this retrieval phase, patients were asked to indicate whether they recognized the presented images from the encoding phases before.

### 2.2. iEEG Recordings and HFO Detection

Invasive EEG recordings were performed via inserted multicontact depth electrodes (AD-Tech; 10 platinum-iridium contacts each). Depth electrodes were inserted from a posterior approach into the hippocampus and rhinal cortex, and electrode locations were documented via post-implantation MRI scans. Furthermore, six patients (patients 1, 4, 5, 6, 8, and 10) received also ECoG (24–102 channels, mean = 45.67) recordings, covering additional temporal lobe areas. In all of these cases, strips covered at least the anterior temporal cortex as well as the lateral temporal cortex. Patients 6 and 10 only received unilateral depth electrode implantations, but had additional large ECoG grids over the respective other hemisphere. In patient 10, depth electrodes were implanted in the left hemisphere and thus could be included in the analyses. In addition to the described invasive EEG recordings, 3–7 scalp electrodes, vertical and horizontal eye movements, an ECG, as well as a facial electromyogram were recorded in each patient during the experiment. Invasive EEG channels were recorded at a sampling rate of 1,000 Hz, and a linked mastoid signal served as reference.

For each patient one encoding and the respective retrieval session were exported to .edf format and then imported to an in-house built software called MEEGIPS (41), for HFO detection. The individual encoding recordings lasted between 305 and 387 s (mean = 329.3 s), whereas the retrieval phase lasted between 903 and 1,011 s (mean = 927 s). On average, HFO analysis was performed on 21 min of EEG data for each participant. The imported EEG data was then analyzed in two ways. First, events of interest were marked visually by one experienced rater, and second, another person conducted an automatic HFO detection.

For visual inspection, the EEG data, as well as additional EMG, ECG, and EOG channels, were prepared in two ways: First, the data was high-pass filtered at 0.1 Hz, and a FIR multiline band reject filter was applied in order to filter out the powerline noise at 50 Hz as well as its respective harmonics. This data was considered the “raw signal.” Second, the data was filtered between 80 and 250 Hz to extract the ripple-band signal, which will be referred to hereafter as the “filtered EEG.” For inspection, both of these signals were displayed next to each other on a screen, and the time cursor was synchronized. Up to eight iEEG channels at a time and the additional EMG, ECG, and EOG channels were visually inspected. In addition to the EEG signals, small windows for the empirical mode decomposition, the discrete Fourier transform, the discrete wavelet power density, and the continuous wavelet transform, calculated from any marked segment of the raw signal, were displayed on the right of the screen. iEEG channels with continuous artifacts corrupting the signal and channels with a generally poor signal-to-noise ratio were excluded.

Ripples were then marked according to the following criteria: (1) consisting of at least four consecutive oscillations both seen in the filtered signal and in the empirical mode decomposition; (2) displaying a regular morphology clearly discernable from the background EEG; (3) revealing an isolated “blob” either in the discrete wavelet power density (DWPD) or in the continuous wavelet transformed signal (CWT; 37); (4) showing a superimposed fast activity in the raw data; and (5) not directly linked to artifacts observed in the EEG, EMG, ECG or EOG channels. Based on these criteria three event categories were identified and marked: (i) ripples, fulfilling all criteria; (ii) unclear HFOs (uHFO), events that did not meet all criteria based on signal quality or unclear evidence of artifacts; and (iii) artifacts, generating ripples meeting the described criteria except the last one. All detected ripples and uHFOs were additionally discussed in the team in order to rigorously exclude all false positive events.

For the automatic detection, the data was decomposed into empirical mode functions (two intrinsic mode functions with a maximum of 100 iterations; 42). Events of interest were detected using an RMS detector with a sliding window size of 10 samples and 1-s-sized statistics segments. The properties for events of interest were fixed as follows: minimum duration of ≥12 ms; RMS transition threshold of 2SD and a peak threshold of 3SD. Events separated by <30 ms were combined taken into account a standard deviation square root. The detected events were then classified based on Stockwell's S-transformation (43) for the frequency range of 80–250 Hz, and a Tukey window was applied to segments 1 s around the center of each event of interest. Events of interest were classified as ripples (autoR) based on a maximum power ratio between the trough and the high-frequency peak of 90%, and a minimum high-frequency to low-frequency peak ratio of 20%. The process of automated HFO detection using these methods has been described in detail by Burnos et al. (44).

### 2.3. Statistical Analysis

The events, detected automatically and visually, were then exported together with the experimental markers and analyzed using MATLAB (release R2019a, The Mathworks, Massachusetts, USA). Rates for all autoR, ripples, uHFO, artifacts, as well as all events detected manually in cumulation were summarized for each encoding and retrieval trial and for each individual patient. Trials were defined as segments starting with stimulus onset and lasting until either patients responded via button press or the next stimulus was presented. In a next step, retrieval trials were paired with their respective encoding trials and grouped into trials with correctly and incorrectly retrieved items (i.e., correct “old” vs. incorrect “new” decisions for previously presented items). Finally, the event rates were related to the respective number of trials and iEEG channels per patient as well as to the lengths of each trial. Thus, we ended up with relative event rates for encoding and retrieval trials corrected for the trial lengths in seconds and for the number of iEEG channels analyzed. For statistical analysis, only events from temporal sites within the hemisphere contralateral to the suspected epileptic zone were considered. In patient 8, we considered the right hemisphere to contain the epileptogenic zone due to the imaging findings, despite seizure onset zones observed in both temporal lobes.

The resulting event matrices were imported into R (45). Statistical analysis aimed at answering three questions to test the general hypothesis. First, we wanted to investigate whether there was a general difference between correct and incorrect trials for the rates of detected events during the retrieval phase. For this purpose, the mean event rates for correct and incorrect trials during retrieval for each patient were entered into a rank-based ANOVA-type test from the package “nparLD” (46) with the two within-subject factors response accuracy (correct vs. incorrect) and event type (all manual events, artifacts, uHFOs, ripples, autoR).

Second, we analyzed whether the rate of events detected during encoding was predictive for the correctness in the subsequent retrieval trials on a trial level. For this purpose, we calculated a generalized linear model with the retrieval trial accuracy as dependent variable and the event rates as predictive factor. Patients were considered as a random factor, in order to take into account variations in baseline events across subjects. Furthermore, we calculated Kendall's correlation between the number of correct trials and the mean event rates per second and estimated a confidence interval using the bias corrected and accelerated bootstrap method with 10,000 bootstrap samples to assess an effect at the group level.

Third, we tested whether the amount of detected events during encoding or retrieval impacted the response time in the retrieval phase. For this we calculated Kendall's correlation between response times and event occurrence rates for each patient individually. We then tested the null hypothesis that the median of these correlations was zero, using a sign test/binomial test: The fact that under the null hypothesis, the number of correlations smaller than zero follows a binomial distribution with probability 0.5 allows for an easy calculation of *p*-values. Correcting for multiple comparisons (13 statistically significance tests) using the Bonferroni method, the adjusted p-threshold was set at 0.0038, in order to avoid an increased family wise error rate.

## 3. Results

**Figure 1:**
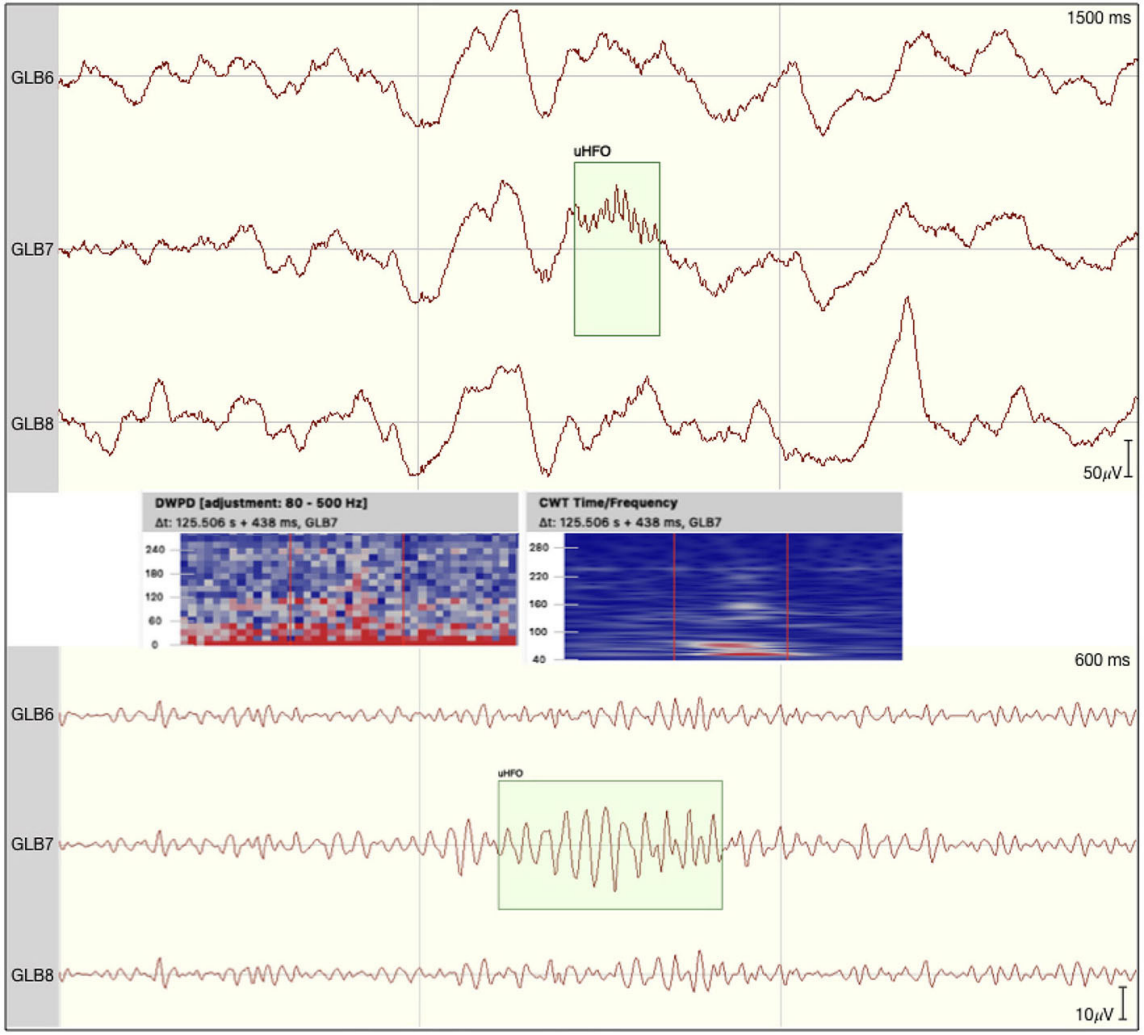
Event of interest defined as unclear HFO by the team as morphology was not regular. Time frequency analyses revealed no clear blob suggesting the potential ripple to be nested in an equally high-frequency noise. The raw signal is depicted at the top with the discrete wavelet power density and the continuous wavelet transformation plotted underneath. At the bottom the signal is filtered between 80 and 250 Hz.

**Figure 2:**
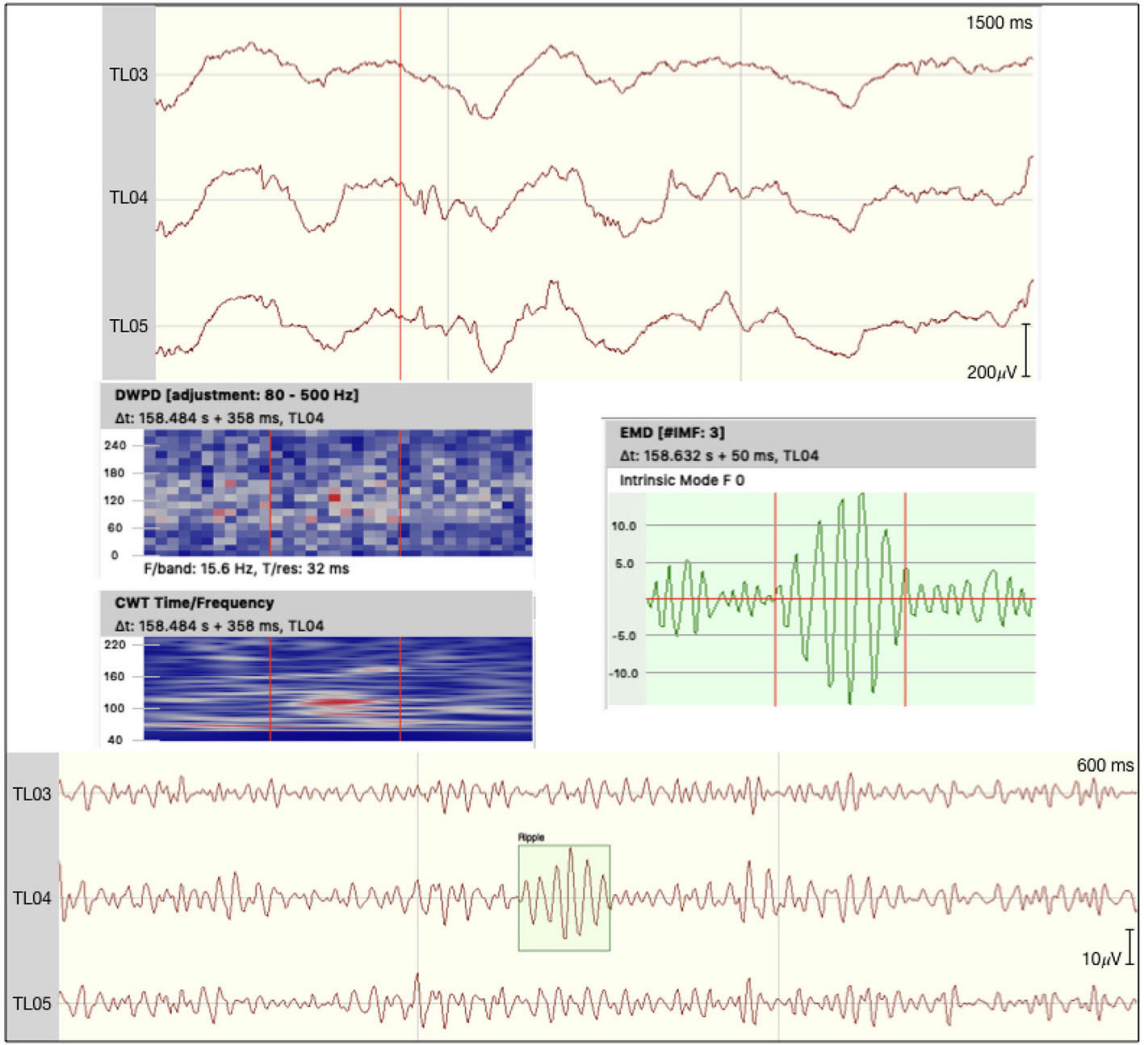
Ripple detected on the epileptogenic site of patient 6. The raw signal is depicted at the top with the discrete wavelet power density, the continuous wavelet transformation, and an empirical mode decomposition (F0) plotted underneath. At the bottom the signal is filtered between 80 and 250 Hz.

All event rates per second for both encoding and the corresponding correct or incorrect retrieval trials are presented in Table 2. No events fulfilled all five criteria to being marked as ripples in the channels of interest. We did detect a small number of unclear HFOs in some of the patients that adhered to most criteria, but could potentially be connected to non-cerebral electrophysiological origins. Figure 1 shows such an uHFO, whereas a clear ripple detected on the ipsilateral site of the suspected epileptic focus in patient 6 is depicted in Figure 2. Notably, not all trials could be taken into consideration, as there were some missing responses during retrieval in patients 1, 2, 5, and 8.

**Table 2:** Event rates per second and iEEG channel, detected during encoding and retrieval in correct (top rows) and incorrect (bottom rows) trials.

**Subject**	**Chans**.	**Trials**	**Resp. time**	**Encoding events/sec**.				**Retrieval events/sec**.			
				**autoR**	**Man. events**	**Artifact**	**uHFOs**	**autoR**	**Man. events**	**Artifact**	**uHFOs**
P1	24	36	1.312	0.657	0.017	0.017	0	0.735	0	0	0
		43	1.428	0.657	0.021	0.021	0	0.682	0.018	0.018	0
P2	10	35	1.208	0.353	0.188	0.188	0	0.376	0.071	0.071	0
		41	1.2	0.261	0.065	0.065	0	0.258	0	0	0
P3	10	28	0.99	0.544	0.089	0.053	0.035	0.711	0	0	0
		28	1.032	0.605	0.051	0.019	0.033	0.696	0.049	0.042	0.007
P4	24	44	0.953	0.535	0.061	0.058	0.004	0.633	0.014	0.014	0
		36	0.943	0.668	0.077	0.069	0.009	0.656	0.023	0.023	0
P5	24	47	0.931	0.247	0.032	0.029	0.003	0.531	0.012	0.012	0
		32	1.101	0.295	0.011	0.01	0.001	0.498	0.024	0.024	0
P6	40	17	0.915	1.048	0.077	0.055	0.022	0.904	0.127	0.122	0.005
		63	0.851	0.992	0.112	0.09	0.023	0.816	0.059	0.057	0.002
P7	10	29	0.965	0.32	0.127	0.127	0	0.55	0.072	0.072	0
		27	0.923	0.362	0.191	0.191	0	0.417	0.04	0.04	0
P8	22	2	0.997	0.521	0	0	0	0.365	0.023	0.023	0
		42	1.152	0.448	0.077	0.077	0	0.512	0.015	0.015	0
P9	10	30	1.044	0.447	0	0	0	0.444	0	0	0
		26	1.082	0.525	0	0	0	0.555	0	0	0
P10	24	17	1.221	0.211	0.124	0.124	0	0.55	0.114	0.144	0
		63	1.139	0.239	0.25	0.25	0	0.37	0.071	0.071	0

*Chans., Nr. of channels; Trials, Nr. of trials; Resp. time, mean response time during retrieval; autoR, automatically detected ripples; man. events, all manually detected events; uHFOs, unclear HFOs.

**Figure 3:**
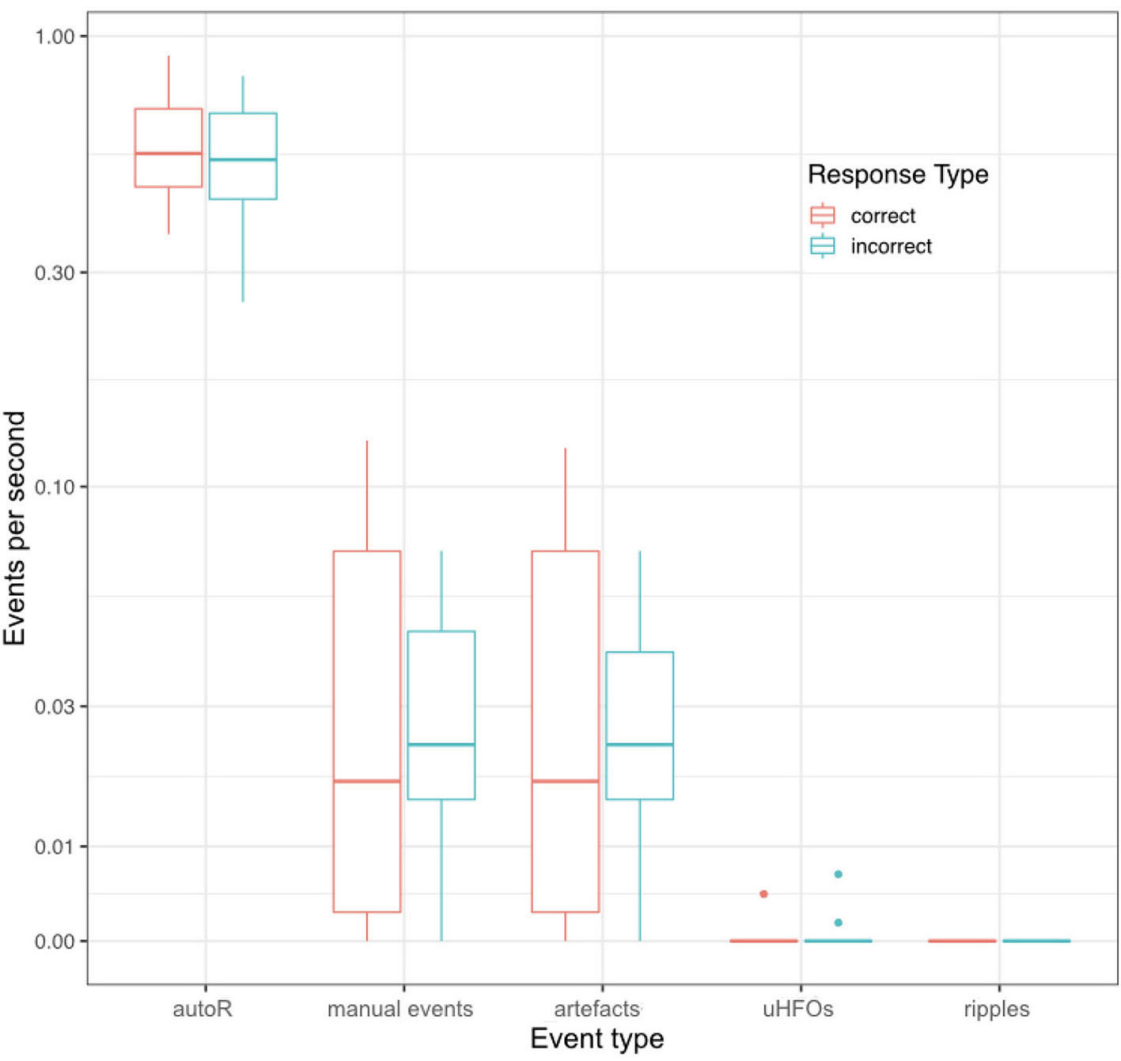
Mean event rates per second during retrieval for all different event types and for correct and incorrect retrieval trials. Each data point corresponds to one patient.

**Figure 4:**
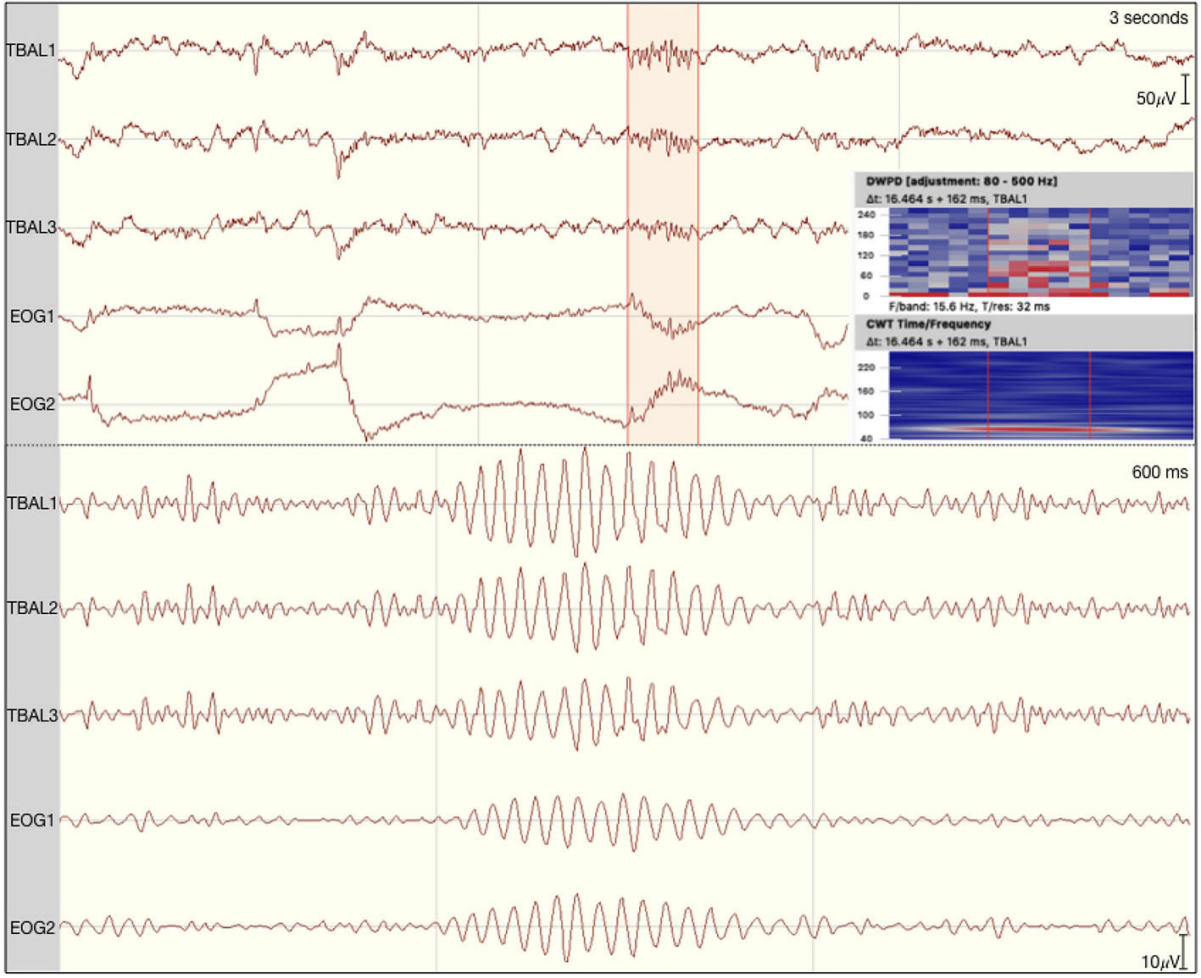
Eye movement-related ripple-like event on the left temporal lobe of patient 1. The raw signal is depicted at the top with the discrete wavelet power density and the continuous wavelet transformation plotted on the side. At the bottom the signal is filtered between 80 and 250 Hz.

Regarding possible differences in event occurrence rates during retrieval, we did not observe a difference between correct and incorrect trials (*F*_1,∞_ = 0.108, *p* = 0.743). There was, however, a main effect for the event type (*F*_1.291,∞_ = 81.514, *p* < 0.001). As can be seen in Figure 3, automatic ripple detection resulted in higher rates across all subjects, regardless of trials being correct or incorrect. Finally, we did not observe an interaction effect between response type and event type (*F*_1.138,∞_ = 0.135, *p* = 0.746). This difference between event types, to some degree, possibly stems from artifactual HFO-like events being marked as ripples. In fact, we have observed plenty of artifacts to mimic HFOs even in the iEEG channels. Especially eye-movements often resulted in such artifactual ripples (see Figure 4 for an example).

**Figure 5:**
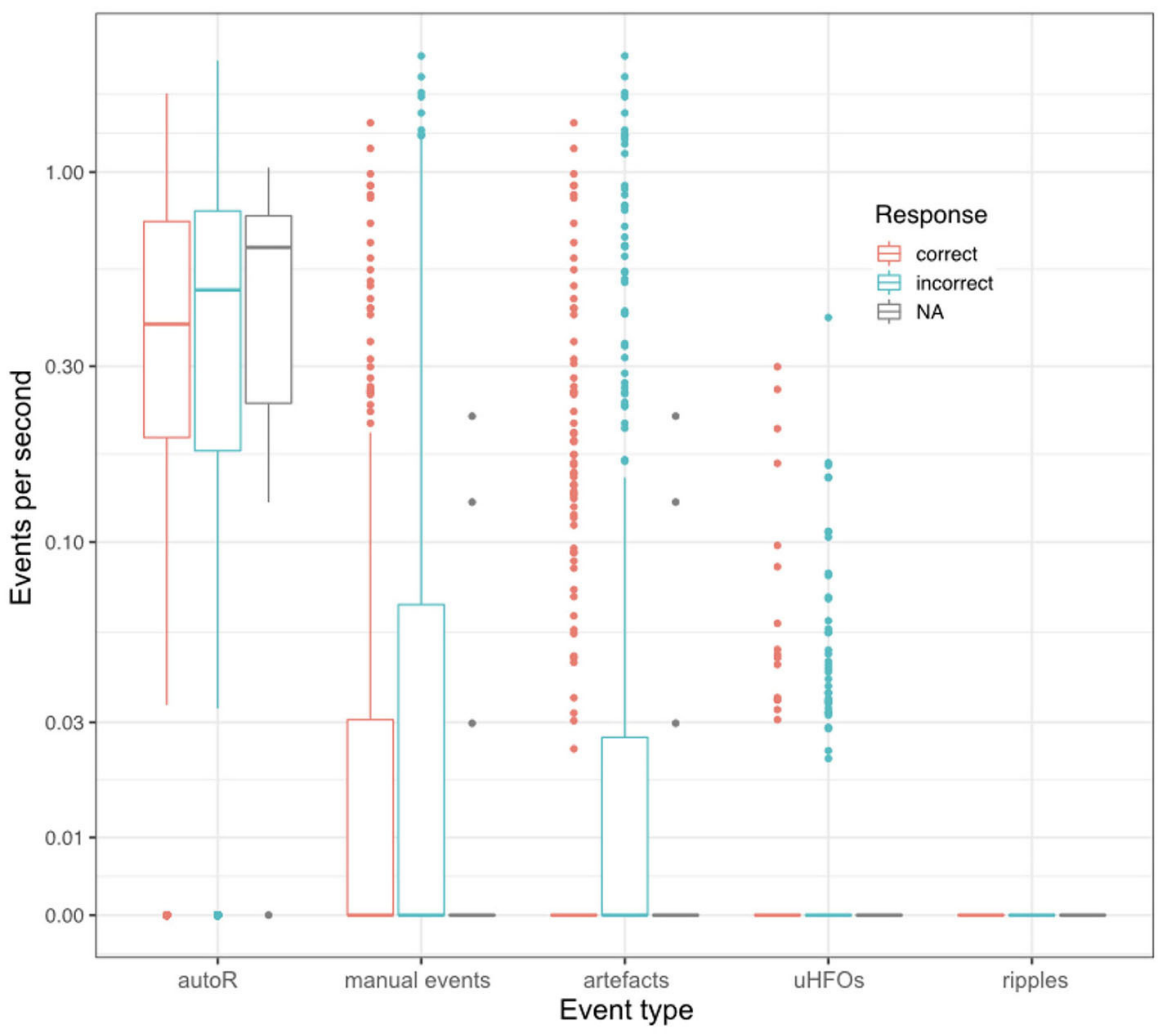
Event rates per second for each encoding trial in relation to the respective response during the corresponding retrieval trial. NA refers to missed responses during retrieval.

**Figure 6:**
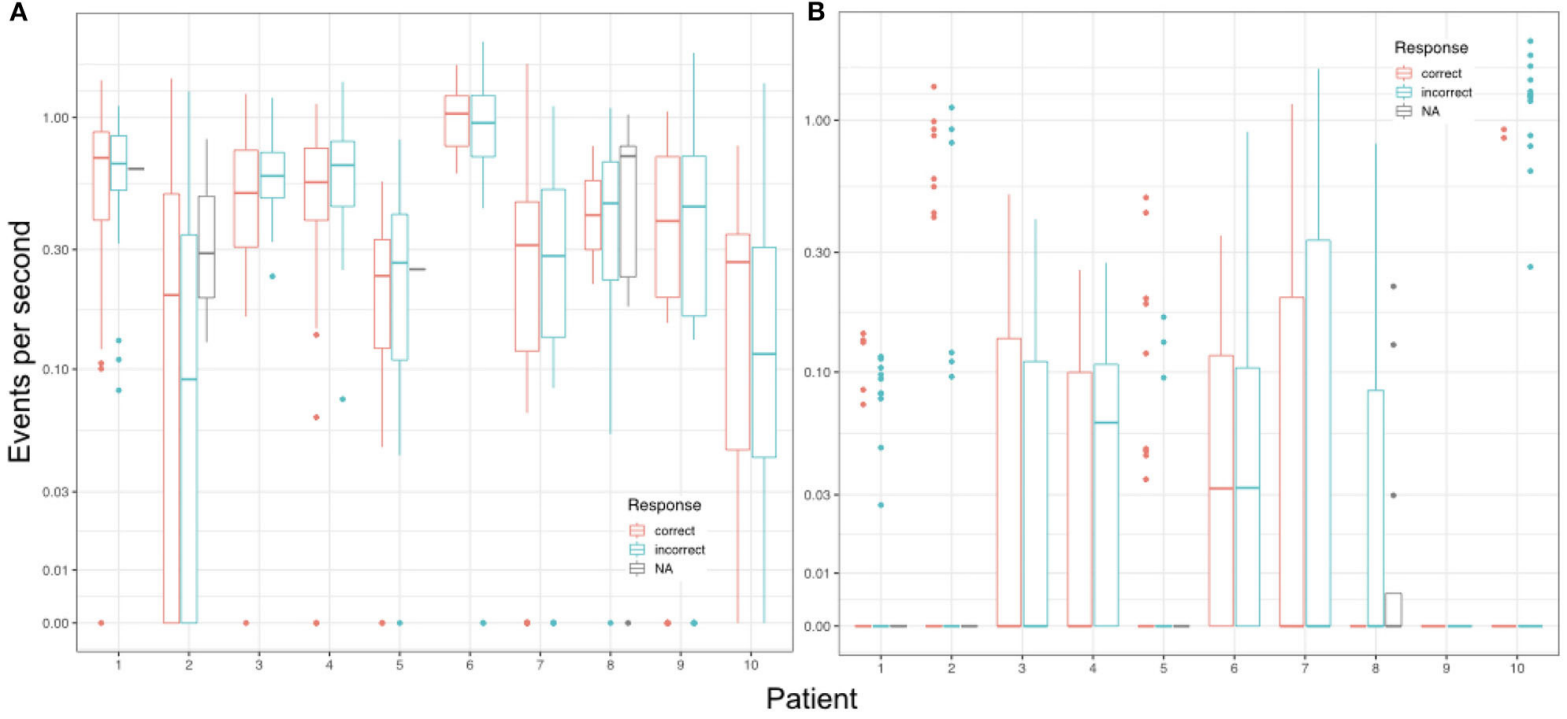
Mean event rates per second during encoding trials in relation to the respective response during the corresponding retrieval trial for each individual patient. **(A)** Shows the results for automatically detected ripples (autoR) and **(B)** depicts all manually detected events. NA refers to missed responses during retrieval.

A higher event rate for automatically detected events was also observed when looking at event rates during encoding. Considering single encoding trials in relation to performance in the corresponding retrieval trials later on, one does not detect an effect for correct vs. incorrect responses (see Figure 5). As such, analysis revealed no predictive values for any of the event types detected during encoding with regards to the later response: autoR (*z* = −0.767, *p* = 0.443), manual events (*z* = −0.515, *p* = 0.607), artifacts (*z* = −0.475, *p* = 0.635), uHFOs (*z* = −0.337, *p* = 0.736). Different baseline rates for patients were taken into account for this analysis, as we expected general differences across patients (see Figure 6). However, in none of the patients the event rate during encoding seemed to affect the response in the respective retrieval trials.

Estimated group correlations between event rates and the number of correct trials corrected for the overall number of trials per patient was weak for all event types. Automatically detected event rates revealed no direction of correlation (τ = 0; CI: −0.684–0.563). Ripple-mimicking artifacts were, however, slightly negatively correlated (τ = −0.27; CI: −0.73–0.15), which also is mirrored in the correlation of all manual events (τ = −0.18; CI: −0.537–0.373). In contrast, uHFOs did not reveal a negative correlation with the number of correct trials (τ = 0.083; CI: −0.462–0.632).

**Figure 7:**
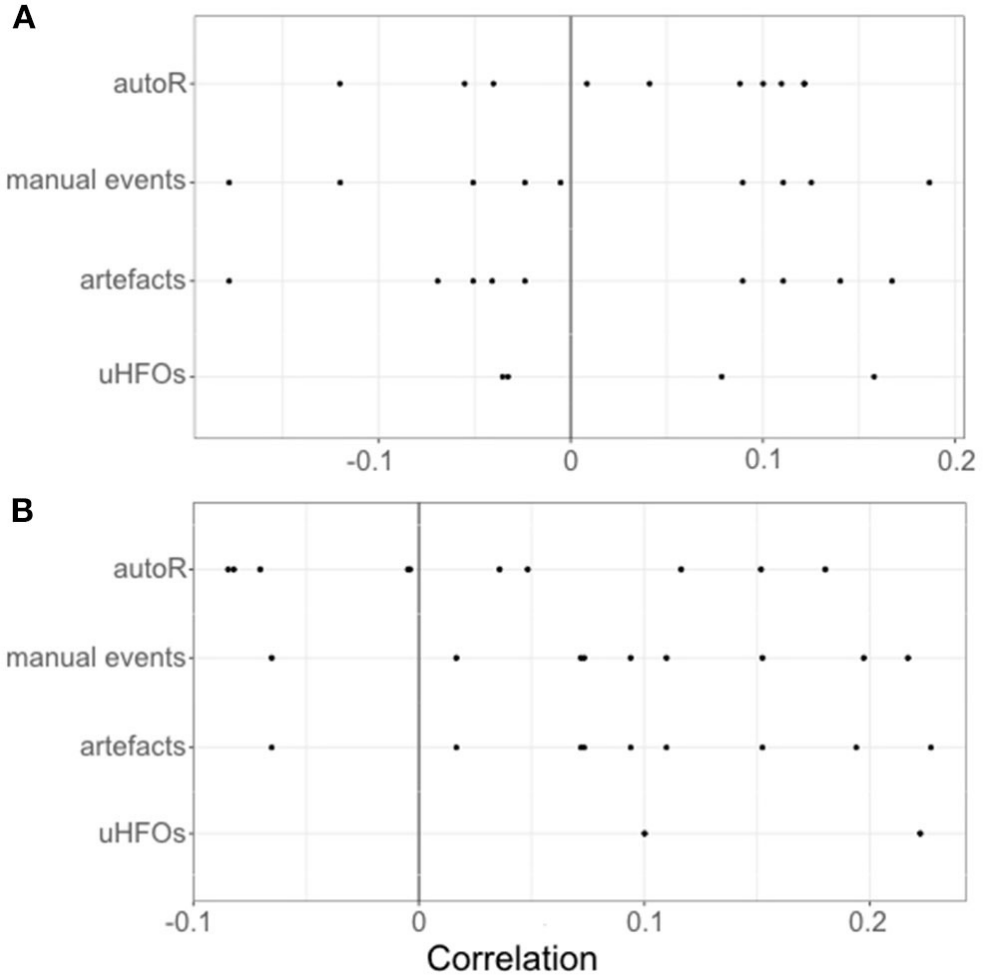
Individual correlations for each patients' event occurrence rates per second during encoding **(A)** and retrieval **(B)** with the response times during the respective retrieval trials.

Finally, we analyzed a relationship between response times and event rates during retrieval trials as well as during the respective encoding trials. The median correlation coefficients for each patient are depicted in Figure 7. Probability testing suggested a trend for retrieval trials with more artifacts and respectively more overall manual events to be longer (both with *Md* τ = 0.094, *p* = 0.039). Other than that, no relationships between event rates and response times for any of the event occurrence rates more extreme than the random binomial probability of 0.5 have been found.

## 4. Discussion

In the present study we aimed at assessing a relationship between the occurrence of stimulus-induced ripples and performance in a visual memory task in order to evaluate two detection approaches for HFOs. We incorporated both, automatic and manual ripple detection and analyzed the iEEG during encoding and retrieval periods of a task, that had previously been reported to induce meaningful HFOs in the resting period between the task phases (20).

Importantly, manual detection did not reveal any events to occur in the iEEG channels contralateral to the suspected epileptogenic zone fulfilling all strict criteria defined for ripples. In contrast, automatic detection revealed significantly higher numbers of events detected in the chosen segments. This discrepancy seems to be caused by a high number of artifacts falsely detected as ripples. In any case, statistical analysis, did not reveal a relationship between task performance and event occurrence rates derived from either detection approach. There was no significant difference between correct and incorrect trials, and also event occurrence during encoding was not predictive of the accuracy in the respective retrieval trials. Furthermore, analyses did not suggest an association between event rates and the time needed to respond during retrieval, either.

In the first part of this section we will elaborate on the incorporated detection strategies, and discuss discrepancies in the detected ripple rates, taking into account important sources of falsely detected events. In the second part, we will briefly discuss physiologic explanations for our findings, especially the lack of manually detected ripple events. Finally, we will consider some limitations to this investigation before drawing an overall conclusion.

### 4.1. Manual vs. Automatic Detection of HFOs

Ever since the first examinations of HFOs, the exact way of detection has left room for debate. The gold standard of visual data inspection and manual marking by one or more raters is highly uneconomic in terms of time and resources needed (12). Furthermore, detecting events that are defined as clearly discernible from the background EEG is subjective, introducing a bias that can be well-appreciated when considering the high variability in events detected by different raters on the same data (29).

Several automatic detection algorithms have been developed to overcome these problems (25, 30–34), making it easier than ever to conduct HFO analyses. However, automatic detection algorithms are not without flaws in their own respect. First of all, algorithms are usually developed and trained on specific data sets, leading to them offering good results in optimal conditions, i.e., a high signal-to-noise ratio and relatively clean data (24, 40). Furthermore, for each algorithm there are numerous settings, that can be altered, making it difficult to compare findings derived from the use of different algorithms and settings.

Second, and more importantly, automatic detection is prone to false-positive detections, resulting from artifacts and sharp transients, that can mimic HFOs after filtering (24, 36, 37), as wells as from a high-frequency noise in the data (24, 35, 38, 39). Even in invasive EEG recordings, which are considered to seldom contain biological artifacts, automatic HFO detection seems to produce a (comparably) high number of false positives.

There have been reports of muscle contractions, body movements and ocular artifacts to corrupt EEG data recorded from deep in the brain (39, 47). Furthermore, eye movements have also been shown to elicit artifacts in brain regions close to extra-ocular muscles (35, 48), appearing as HFO-like events. In line with these reports, we also found ripple-like events to coincide with eye-movements and, when filtered, EOG revealed similar HFO-like derivates as iEEG channels, suggesting eye movement-related ripples to also appear in the iEEG. Taking into account additional channels, such as EOG and EMG, highly increased the number of events defined as artifact-derived HFOs in our data.

Comparing both detection approaches, manual detection led to only few events being considered as possible ripples in our data. Taking into account additional channels, such as EOG and EMG, highly increased the number of events defined as artifact-derived HFOs. Considering these additional channels may be crucial when opting for HFO detection, even in intracranial EEG data. While the strict visual detection led to a high specificity, automatic detection appeared to produce a very high number of false positives. These findings underline the pitfalls of automated HFO detection. Preprocessing the data with special emphasis on reducing artifacts or training algorithms to acknowledge artificial HFOs might prove helpful to increase the specificity of detection algorithms (49, 50).

Given the lack of visually detected clear ripples, and the extreme discrepancy between the detection approaches, further point to a need for a more precise definition of what truly constitutes an HFO. While a very strict definition, as applied in our manual detection, leads to very few or even no clear HFOs to be detected, it may serve as a basis to align detection strategies between different rater, research groups, and different detection algorithms. Besides, the methodological and technical interpretation of our findings, there are also some physiologic explanations for the lack of manually detected ripples in our data.

### 4.2. Memory Task-Related HFO Occurrence

Neither of the two incorporated HFO detection approaches yielded event rates, that could be linked to performance during encoding or retrieval in the visual memory task. It should be noted, however, that we correlated event rates across all analyzed channels with memory performance and did not subselect specific channels. Furthermore, we were unable to manually detect clear ripples in the data. This finding is notable, given the numerous notions of spontaneous HFO occurrence in memory-related brain areas (3, 14, 15, 20). One explanation for the incompatible findings could be that our manual detection criteria were extremely strict (maybe too strict) and missed physiologic ripples that did not conform to the ideal pattern. Another explanation could lie in the fact, that these studies all investigated HFOs during periods of rest and sleep.

Sleep has been suggested to offer a unique window into memory consolidation via hippocampal reactivation (18, 51–53), and thus might offer an increased probability to record memory-related HFOs. Especially hippocampal ripples being nested in sleep spindles have been suggested to be crucial for long-term potentiation and memory consolidation (22, 54, 55). Furthermore, resting and sleep EEG may provide data with a higher signal-to-noise ratio. Especially, high background noise and artifacts, that might have also been induced by the task, can lead to a number of false-positives for automatic detectors (24, 56). This would explain the discrepancy between the automatic and manual detection, as visual inspection would not have considered events embedded within a noisy background.

On another note, continuous high frequency activity in the background EEG has been suggested to reflect physiological activity distinctive for certain brain regions (57). This is in line with reports of high gamma band activity (including frequencies that fall into the ripple band) being related to memory (58, 59). These studies further point to a weakness in detecting single HFO events, as ripple band activity might be not only easier to detect during memory tasks, but also reveal important links to memory processes. Thus, a shift in focus from single oscillatory events to frequency band characteristics when studying cognition may be promising. Distinguishing HFOs from high frequency activity in this context may have the further benefit of ruling out epilepsy-related HFOs confounding the events of interest (60, 61).

### 4.3. Limitations

There are some limitations to the study at hand, some of which have already been outlined in the discussion. First, performing a manual detection with one rater only may result in very stable event detections across recordings, however multiple raters might have increased the sensitivity of visual detection. Since all unclear events and marked ripples were discussed in the team, specificity would not have changed with multiple raters, though. Second, differences between the two detection strategies have to be interpreted with caution, bearing in mind that we chose two very extreme approaches. The visual detection was performed strictly, with events of interest only being marked as ripples in case of no doubt. In contrast, the automatic detection algorithm's settings were chosen to increase sensitivity in order to make the differences between both detection strategies as visible as possible.

Third, the external reference used (linked mastoids) may have contributed to the artifact contamination of our iEEG data. The impact of the reference electrodes have already been described, and to this end a bipolar montage might have resulted in less artifactual events (24, 35, 62), which would have impacted the data for both detection strategies, however. Finally, numbers of trials between patients differed, especially with respect to correct and incorrect trials. Thus, the statistical sample was small for some analyses. This fact in connection with the small number of events for some types likely led to a low statistical power, which even carefully selected statistical tests may not have been able to compensate. Regardless of these limitations, there are some conclusions that can be drawn from the obtained results.

### 4.4. Conclusion

Our findings suggest grave differences between automatically and manually detected events. Our analysis suggests automatic detection to be highly affected by false ripples derived not only from technical but also from physiologic artifacts. Recording additional facial EMG as well as EOG channels seems beneficial for the identification of false ripples even in iEEG data. Future automated detection algorithms should implement artifact matching in these additional channels, in order to improve specificity. Also developing a preprocessing pipeline in order to clean the data of artifacts before automatic algorithms detect HFOs could be a potential aim for future studies. Until then, guidelines for a more strict and careful visual inspection are needed to ensure comparable results, especially when dealing with conditions that seldom offer ideal data, for instance when performing cognitive paradigms.

Finally, we were not able to visually detect clear ripples, and other event types, including automatically detected ripples, could not be related to memory processes. Therefor, it remains questionable whether HFOs as single events can be exclusively identified as physiologic biomarkers. For now high frequency activity rather than single high frequency events may present a more suitable surrogate marker for cognition. Being also less affected by epileptogenity as well as artifacts, it is also less time-consuming to investigate high frequency band activity, thus offering another promising approach for future studies.

## Data Availability Statement

The raw data supporting the conclusions of this article will be made available by the authors, without undue reservation.

## Ethics Statement

The studies involving human participants were reviewed and approved by local ethics committee: Ethikkommission an der Medizinischen Fakultät der Rheinischen Friedrich-Wilhelms-Universität Bonn, Venusberg Campus 1 (Geb. 02), 53127 Bonn. The patients/participants provided their written informed consent to participate in this study. Written informed consent was obtained from the individual(s) for the publication of any potentially identifiable images or data included in this article.

## Author Contributions

AT wrote the initial manuscript and performed the automatic event detection. NG manually detected the events and developed the marking strategy in correspondence with AT and YH. PL performed the marker statistics supervised by AB. JF prepared the EEG data for analysis and conducted the initial study. ET and YH supervised the event detection strategies and general data analysis. All authors read and critically revised the manuscript before submission.

## Conflict of Interest

The authors declare that the research was conducted in the absence of any commercial or financial relationships that could be construed as a potential conflict of interest.
